# Improvement in Symptoms of Depression and Anxiety and Cardiometabolic Risk Factors in Children and Adolescents with Overweight and Obesity Following the Implementation of a Multidisciplinary Personalized Lifestyle Intervention Program

**DOI:** 10.3390/nu16213710

**Published:** 2024-10-30

**Authors:** Aikaterini Vourdoumpa, George Paltoglou, Maria Manou, Emilia Mantzou, Penio Kassari, Marina Papadopoulou, Gerasimos Kolaitis, Evangelia Charmandari

**Affiliations:** 1 Division of Endocrinology, Metabolism and Diabetes, First Department of Pediatrics, National and Kapodistrian University of Athens Medical School, ‘Aghia Sophia’ Children’s Hospital, 11527 Athens, Greece; katvourdouba@gmail.com (A.V.); mariamanou93@hotmail.com (M.M.); amantzou@med.uoa.gr (E.M.); peniokassari@gmail.com (P.K.); marinageorpap@gmail.com (M.P.); 2 Diabetes and Metabolism Clinic, Second Department of Pediatrics, National and Kapodistrian University of Athens, “P. & A. Kyriakou” Children’s Hospital, 11527 Athens, Greece; gpaltoglou@gmail.com; 3 Center of Clinical, Experimental Surgery and Translational Research, Biomedical Research Foundation of the Academy of Athens, 11527 Athens, Greece; 4 Department of Child Psychiatry, National and Kapodistrian University of Athens Medical School, ‘Aghia Sophia’ Children’s Hospital, 11527 Athens, Greece; gkolaitis@med.uoa.gr

**Keywords:** childhood obesity, mental health, depression, anxiety, lifestyle intervention

## Abstract

**Background/Objectives**: Childhood obesity is one of the most challenging contemporary public health problems. Children and adolescents with obesity experience multiple psychosocial difficulties, such as low self-esteem, depression, anxiety, and behavioral problems, which persist for a long time. The aim of the study was to assess the effect of a multidisciplinary personalized lifestyle intervention for depressive and anxiety symptoms, as evaluated by psychometric questionnaires, and their effect and association with cardiometabolic parameters in children and adolescents with overweight and obesity before and after the intervention. **Methods**: Six hundred and eleven (n = 611) children and adolescents (mean age ± SE: 10.39 ± 0.10 years; 51.5% females, 46.6% pubertal) were studied prospectively. Subjects were classified as being obese (50.2%), overweight (33.5%), or having a normal BMI (16.2%) according to IOTF criteria. All participants entered a 1-year lifestyle intervention program; laboratory investigations were obtained at the beginning and end of the study and two psychometric questionnaires were completed, the CDI and SCARED, which evaluate symptoms of depression and anxiety, respectively. **Results**: Following the lifestyle intervention, a significant decrease was noted in anxiety scores in all subjects and in depression scores in youth with obesity, as well as in adolescents with obesity, while females displayed a reduced response to the intervention. Insulin resistance and metabolic syndrome parameters, cortisol, PRL, and LH concentrations were positive predictors for depressive and anxiety symptoms. **Conclusions**: The implementation of a multidisciplinary personalized lifestyle intervention program in the management of childhood obesity is associated with a significant decrease in cardiometabolic and psychosocial comorbidities in children with and without excess adiposity. The improvement in mental health is likely mediated by an improvement in energy metabolism with subsequent improvement in neuroinflammation owing to lifestyle changes.

## 1. Introduction

Childhood obesity has arisen as one of the most challenging public health problems of our century [1]. In Europe, the highest rates are recorded in Mediterranean countries, with Greece having a leading role [2]. Indeed, the prevalence of overweight and obesity in Greece ranges from 21% in preschool-aged children to 41% in school-aged children and adolescents, and it is significantly higher than the rest of the European countries (15% and 25%, respectively) [3]. Childhood obesity constitutes a multifactorial chronic disease caused by the synergistic action of multiple genes, epigenetic mechanisms, and environmental factors, with genome penetrance being the highest during childhood [4,5]. Multiple comorbidities emerge, including cardiovascular and metabolic disorders, as well as psychosocial disorders, such as social stigma, low self-esteem, and behavioral problems [6,7]. Children and adolescents with excess adiposity have a five-fold risk of having obesity in adulthood [8], while cardiometabolic risk is independently associated with serious comorbidities later in life, including diabetes mellitus type 2, hypertension, coronary artery disease, and dyslipidemia, as well as increased mortality [9,10,11,12,13].

Interestingly, youth with excess adiposity are at an increased risk of developing anxiety and depression, which constitute prevalent psychosocial disorders in this population and persist over time [7,14]. Childhood and adolescence constitute a developmental milestone in which individual, social, and emotional habits that are of paramount importance for long-term well-being are developed, such as adopting healthy sleep patterns, regular exercise, developing coping and problem-solving skills, and managing interpersonal relationships and emotions [15]. Τhe physical and psychological changes combined with the constant social challenges (poverty, abuse or violence, domestic and interpersonal problems, the COVID-19 pandemic, addictions, and chronic diseases) that occur during this evolutive age make youngsters particularly affected by the global obesity epidemic and prone to anxiety, depression, and unhealthy lifestyle patterns [16,17]. Alcohol and illicit substance abuse present a worrisome trend, with about 19.9% of 14- to 15-year-old adolescents reporting having at least one drink in their lifetime and at the same time, 10.9% of eighth graders, 19.8% of 10th graders, and 31.2% of 12th graders using any illicit drug in 2023, according to the National Survey on Drug Use and Health (NSDUH) [18].

The influence of endogenous biological factors derived from changes in energy metabolism is significant on the development of anxiety and depression in individuals with excess adiposity [19]. Growing evidence highlights the bidirectional relationship between diets high in saturated fat and sugar, a sedentary lifestyle and visceral fat accumulation, and the psychological consequences of obesity. Obesity-induced metabolic and vascular dysfunction, including chronic gut and adipose tissue inflammation, insulin and leptin resistance, and arterial hypertension, as well as dysregulation of the hypothalamic–pituitary–adrenal (HPA) axis, have emerged as the key pathophysiological mechanisms for the development of depression and anxiety in individuals with obesity. The inflammatory state of obesity affects neuroimmunological responses and the function of neuronal circuits (dopaminergic and serotonin pathways) in the central nervous system, leading to neuroinflammation and altering the structure, excitability, and connectivity of neuronal networks that control mood, motivation, and emotion. The interplay between metabolic and mood disturbances can perpetuate a cycle of despair, overeating, and physical inactivity, which reinforces the severity of obesity and its comorbidities [19]. Indeed, dopaminergic pathways are enhanced by pleasurable stimuli (palatable food, drugs, alcohol) in prone young individuals, providing greater reward value, especially in the presence of risk factors and disrupted interpersonal relationships [20].

The Center for Disease Control and Prevention (CDC) emphasizes the supportive role of a healthy lifestyle [21]. A healthy eating pattern focusing on fruits, vegetables, whole grains, legumes, lean sources of protein, and nuts and seeds, as well as participation in physical activity for at least 60 min each day, getting sufficient sleep each night, and practicing mindfulness or relaxation techniques, are behaviors that address excess adiposity and help to promote mental health. Overall, it is imperative that childhood obesity is prevented and managed early prior to the onset of physical or mental comorbidities [7].

The aim of the present study was to study the effect of a comprehensive, multidisciplinary, and personalized lifestyle intervention program for depressive and anxiety symptoms, as evaluated by psychometric questionnaires (CDI and SCARED, respectively), and their effect and association with cardiometabolic parameters in children and adolescents with overweight and obesity before and after the intervention. The main research hypothesis was that the improvement in BMI following the implementation of the lifestyle intervention program in youth with or without excess adiposity was associated with an improvement in cardiometabolic risk factors and anxiety and depressive symptomatology.

## 2. Materials and Methods

### 2.1. Study Design

We conducted a prospective study of children and adolescents, who were consecutive our Center for the Prevention and Management of Overweight and Obesity in Childhood and Adolescence, Division of Endocrinology, Metabolism and Diabetes, First Department of Pediatrics, National and Kapodistrian University of Athens Medical School, ‘Aghia Sophia’ Children’s Hospital. A structured, comprehensive, multidisciplinary, and personalized lifestyle intervention program was implemented for 1 year and data were collected both at the initial and annual assessment. The study was conducted in accordance with the principles of the Declaration of Helsinki and was approved by the Committee on the Ethics of Human Research of ‘Aghia Sophia’ Children’s Hospital (approval number: EB-PASCH-MoM: 28 November 2013, ref: 10290-14/05/2013 and approval number: EB-PASCH-MoM: 3 April 2018, ref: 7000-20/03/2018). Parents or legal guardians provided written informed consent in all cases and children ≥ 7 years gave their assent. Children and adolescents not participating in the study received equal healthcare.

### 2.2. Patients

From a total of 708 children and adolescents of 6–18 years of age who were initially recruited to participate in the study, 611 participants completed the 1-year lifestyle intervention (13.7% drop-out rate). The six hundred and eleven [n = 611, mean age ± SE: 10.39 ± 0.10 years; 315 (51.5%) females, 296 (48.5%) males] subjects were classified as being obese (n = 307, 50.2%), overweight (n = 205, 33.6%), or having a normal BMI (n = 99, 16.2%) according to the International Obesity Task Force (IOTF) criteria [22,23,24] and as prepubertal (n = 326, 53.4%) or pubertal (n = 285, 46.6%) according to the Tanner pubertal assessment. The clinical characteristics and laboratory investigations of all participants are presented in Table 1 and Table S1.

### 2.3. Study Protocol: Initial Assessment, Intervention, and Annual Follow-Up

All participants were admitted to our endocrine unit early in the morning of their first visit. A detailed medical history and thorough clinical examination was performed, and standard anthropometric measurements were obtained by trained clinicians. Baseline blood samples for hematologic, biochemical, and endocrinologic investigations were obtained at 08:00 h after a 12 h overnight fast. All participants entered the 1-year multidisciplinary lifestyle intervention program for the management of overweight and obesity that provided personalized advice and guidance on healthy diet, sleep, and physical activity to both patients and their families, as previously described in detail [25,26,27,28]. A pediatrician, pediatric endocrinologist, pediatric dietician, a professional fitness personal trainer, and—if necessary—a pediatric psychologist evaluated all subjects at baseline and at frequent intervals thereafter.

The protocol of the lifestyle intervention included monthly follow-up assessments for subjects with obesity, two-monthly assessments for subjects with overweight, and three-monthly assessments for subjects with a normal BMI. During each follow-up visit, participants were evaluated by the multidisciplinary team and all anthropometric parameters were measured; a clinical evaluation, including Tanner pubertal staging, was performed, a 24 h diet recall was recorded, and the goals set at previous visits were analyzed in detail. Upon the completion of the study 1 year later, detailed hematologic, biochemical, and endocrinologic investigations were performed again at 08:00 h following a 12 h overnight fast.

More specifically, the pediatric dietician evaluated the daily nutritional patterns of all participants and performed a 24 h recall according to the USDA method [29]. In this way, a personalized plan of a healthy diet was created, which was based on individual preferences, food availability, whether food was bought or prepared at school or at home, and everyday life habits. All subjects were advised to eat three main meals (breakfast, lunch, and dinner) and two healthy snacks (fruits, vegetables) at mid-morning and mid-afternoon every day, according to the “My Plate” standard, a visualized approach of the 2010 USDA guidelines [30], and the guidelines proposed by the National Nutritional Guide for Infants, Children, and Adolescents [31]. The personal trainer recorded the weekly hobbies and activities of all subjects and discussed the child’s preferences with the family in order to set a personalized physical activity program aimed to be perceived as highly enjoyable and entertaining. In addition, the whole family was encouraged to avoid a sedentary lifestyle and to engage in daily physical activities of their choice for 30–45 min, such as walking, cycling, and dancing. Finally, when necessary, a pediatric psychologist further evaluated subjects in need and their families at frequent intervals and referred them to a pediatric psychiatrist if needed. Detailed recommendations for adequate sleep were also provided to each participant based on their age, according to the American Academy of Sleep Medicine Consensus Guidelines [32]. These guidelines suggested 9 to 12 h of sleep per day for children aged 10–12 years, and 8 to 10 h per day for adolescents aged 13–18 years. Participants were advised to prioritize uninterrupted sleep, aiming to fall asleep as early as possible before midnight and maintain a consistent sleep schedule every day. Also, children were advised to limit screen time to less than two hours per day and to turn off all electronic devices one hour before bedtime.

Compliance was assessed at the annual evaluation based on attendance to the follow-up appointments, with all included subjects completing the study and attending all follow-up visits with the multidisciplinary team for clinical, laboratory, and psychometric evaluations and advice on diet, sleep, and exercise, thus providing a “good compliance” with the protocol.

### 2.4. Anthropometric and Body Composition Parameters

The assessment of body weight was conducted in light clothing and without shoes using the same scale for all subjects (Seca GmbH & Co. KG., Hamburg, Germany). Standing height was also measured without shoes using a Harpenden’s stadiometer (Holtain Limited, Crymych-Dyfed, UK). The BMI was expressed as weight in kilograms (kg) divided by height in meters squared (m2), and the BMI z-score was calculated according to the Greek standard growth charts [33]. The WHO STEPwise approach to surveillance (STEPS) protocol was implemented to measure waist and hip circumferences using the same stretch-resistant tape (Seca GmbH & Co. KG., Hamburg, Germany) with the subjects in a standing position [34]. Systolic (SBP) and diastolic blood pressure (DBP) were measured twice by a sphygmomanometer (Comfort 20/40, Visomat, Parapharm, Metamorphosi, Attiki, Greece) using a suitable-for-age cuff, and the mean value was calculated.

### 2.5. Assays

The hematologic investigations were performed using the ADVIA 2110i analyzer (Roche Diagnostics GmbH, Mannheim, Germany). Glucose, total cholesterol, triglycerides, and high-density lipoprotein (HDL) cholesterol were determined using the ADVIA 1800 Siemens analyzer (Siemens Healthcare Diagnostics, Tarrytown, NY, USA). Hemoglobin A1C (HbA1C) was determined through reversed-phase cation exchange high-performance liquid chromatography (HPLC) on an automated glycohemoglobin analyzer HA-8160 (Arkray, Kyoto, Japan). Apolipoproteins A1 (Apo-A1) and B (Apo-B) and lipoprotein a (Lpa) concentrations were measured by means of latex particle-enhanced immunonephelometric assays on the BN ProSpec nephelometer (Dade Behring, Siemens Healthcare Diagnostics, Liederbach, Germany).

Follicle-stimulating hormone (FSH), luteinizing hormone (LH), estradiol, ferritin, and insulin concentrations were measured using an automated electrochemiluminescense immunoassay analyzer (Cobas e411, Roche Diagnostics GmbH, Mannheim, Germany). Thyroid-stimulating hormones (TSHs), triiodothyronine (Τ3), free thyroxine (FT4), antithyroid peroxidase antibodies (anti-TPOs), anti-thyroglobulin antibodies (anti-TGs), adrenocorticotropin (ACTH), cortisol, androstenedione, testosterone, dehydroepiandrosterone sulfate (DHEAS), insulin-like growth factor-I (IGF-I), and insulin-like growth factor-binding protein-3 (IGFBP-3) concentrations were calculated by chemiluminescence immunoassays on an IMMULITE 2000 immunoassay system (Siemens Healthcare Diagnostics Products Ltd., Surrey, UK). The total 25-hydroxyvitamin D (25-OH-vitamin D) concentration was determined using an automated electrochemiluminescence immunoassay on the Modular Analytics E170 analyzer (Roche Diagnostics, Basel, Switzerland).

The homeostasis model assessment (HOMA-IR), as a measure of insulin resistance, was determined as follows: HOMA-IR = [fasting glucose (mg/dL) × fasting insulin (mU/L)]/405.

### 2.6. Psychometric Questionnaires 

To detect symptoms of depression and anxiety, all participants were requested to fill out two psychometric questionnaires, the Children’s Depression Inventory (CDI) and the Screen for Child Anxiety-Related Disorders (SCARED), both at the initial and annual assessment.

#### 2.6.1. Children’s Depression Inventory (CDI)

The CDI questionnaire is one of the most widely used and best studied scales worldwide for the identification of depressive symptomatology in children and adolescents 6–18 years old [35,36]. The CDI evaluates essential features of depression in accordance with the DSM-V [37]. Symptoms of depression are assessed through self-reported questions, creating five factor subscales (negative mood, interpersonal difficulties, ineffectiveness, anhedonia, negative self-esteem), which add to a total scale [38]. The child is asked to answer 27 Likert-type items with 3 provided statements regarding his/her feelings in the last two weeks [38]. Each statement is scored from 0 to 2, quantitively reflecting a growing severity of symptoms [38]. The CDI was successfully standardized for the Greek population with an internal consistency (Cronbach’s alpha) of 0.805 and test–retest reliability > 0.60, and the cut-off point of score 15 (90th percentile) was set as a screening threshold [39].

#### 2.6.2. Screen for Child Anxiety-Related Disorders (SCARED)

The SCARED is an instrument for screening anxiety symptomatology in childhood and adolescence, which is well established in different countries worldwide [35,40]. It is a self-reported questionnaire designed for both children and adolescents 6–18 years old and their parents, evaluating the presence of symptoms in youngsters for the last 3 months [41]. The long version of the SCARED consists of 41 Likert-type questions, which create five subscales (panic/somatic, generalized anxiety, separation anxiety, social phobia, school phobia) [41,42] compatible with the categorization of the DSM-V [28] for anxiety symptomatology. A shorter five-item version has also been created, consisting of questions with a higher discriminant function in each subscale [41]. The latter shorter version was used in the present study. Each item is scored from 0 (never) to 2 (often or always), adding to a final score [41]. Three out of ten is considered the screening threshold [41]. The SCARED has been used in the Greek language successfully [43,44].

### 2.7. Statistical Analysis

All analyzed variables followed a normal distribution and the reported results are presented as mean ± standard error of the mean (SE). Significant effects were revealed in the LSD post hoc test and statistical significance was set at *p* < 0.05. The comparison of variables between the initial and annual assessment was performed by employing repeated-measures analysis of variance (ANOVA) tests, Chi-square analysis, and Yates correction. Potential predictors of anxiety and depressive symptomatology were explored through standard forward stepwise multiple linear regression models. All statistical analyses were performed with Statistica 8 software (StatSoft, Tulsa, OK, USA).

## 3. Results

Six hundred and eleven (n = 611) children and adolescents [mean age ± SE: 10.39 ± 0.10 years, females: 315 (51.5%), males: 296 (48.5%), prepubertal: 326 (53.4%), pubertal: 285 (46.6%)] participated in the 1-year personalized multidisciplinary lifestyle intervention program. Participants were classified as being obese (n = 307, 50.2%), overweight (n = 205, 33.6%) or having a normal BMI (n = 99, 16.2%) according to the International Obesity Task Force (IOTF) criteria [22,23,24]. Table 1 presents the clinical characteristics, anthropometric parameters, hematologic, biochemical, cardiometabolic risk factor, and endocrinologic investigations, as well as psychometric scores, both at the initial and annual assessment, and the correlations among them.

### 3.1. Clinical Characteristics, Anthropometric Parameters, Hematologic, Biochemical, Cardiometabolic Risk Factor and Endocrinologic Investigations and Psychometric Questionnaires Scores

Subjects with obesity had a significantly increased SBP, DBP, waist circumference (WC), hip circumference (HC), waist-to-hip ratio (WHR), and waist-to-height ratio (WHtR) at the initial and annual assessments compared to their overweight and normal BMI counterparts. Moreover, inflammatory makers [white blood cells, erythrocyte sedimentation rate (ESR), ferritin], insulin sensitivity parameters (insulin concentration, HbA1C, HOMA-IR), and cardiometabolic risk factor parameters (uric acid, triglycerides, LDL, Apo-B) were significantly higher, while HDL, Apo-A, and 25-OH-vitamin D concentrations were significantly lower in subjects with obesity compared to those with overweight and normal BMI.

After the implementation of the 1-year lifestyle intervention program, there was a significant reduction in body weight (Wt), BMI, BMI z-score, WC, and WHtR in participants with obesity and overweight compared to the initial assessment. In addition, there was a significant improvement in the inflammatory markers (ESR, ferritin), HbA1C, cardiovascular risk factors (LDL, HDL, ApoA1, AST, ALT, γ-GT, uric acid), and 25-OH-vitamin D concentration. Moreover, increases in hormonal factor concentrations were observed (IGF-I, IGBP3, Δ4, testosterone, DHEAS, PRL, LH, FSH, E2), as well as a reduction in thyroid hormones (Τ3, fT4) with our analysis using the data combined for both sexes.

To begin with, participants were categorized based on the questionnaires’ threshold for the detection of pathology into two groups (pathological and normal score), using the score as a categorical variable. At the initial assessment, depressive symptom scores were present in 13%, 13.2%, and 9.1% of participants with obesity, overweight, and a normal BMI, respectively. Pathological scores indicating anxiety symptoms were present in 34.9%, 34.1%, and 39.4% in SCARED children scores and 23.5%, 23.9%, and 22.2% in SCARED parent scores in subjects with obesity, overweight, and a normal BMI, respectively. No statistically significant associations were observed between the BMI category and normal or pathological scores at the initial or annual assessments (Table S2).

Furthermore, the effect of the lifestyle intervention on the change in psychometric scores was examined, using scores as a quantitative variable (Table 1 (F)). Children and adolescents with obesity demonstrated a statistically significant improvement in CDI scores after the implementation of the 1-year lifestyle intervention program compared to the initial assessment. Meanwhile, a statistically significant decrease was also observed in SCARED children scores in children and adolescents with obesity, as well as in all subjects. Furthermore, a significant amelioration in SCARED parent scores in all BMI groups was noted. The effect of the intervention on psychometric scores was further investigated, using scores as a categorical variable based on the threshold of the questionnaires for detecting pathology in two groups, namely the pathological and normal scores (Table 2). After the intervention, there was a significant improvement in pathologic scores in the CDI and SCARED scores in all BMI groups and in the whole sample, except for anxiety scores in the normal BMI group in the SCARED based on the children’s perspective.

The sample was further categorized by gender (Table S3). At baseline, males with obesity demonstrated higher anxiety and depressive symptom scores compared to their male counterparts with a normal BMI. An improvement in depressive symptom scores after the intervention was observed in males with obesity compared to the initial assessment, a correlation that was not observed in the female gender. Gender did not affect other associations.

When participants were categorized based on pubertal stage (Table S4), pubertal subjects with obesity had significantly higher CDI scores compared with prepubertal subjects with obesity at the initial assessment. At the annual assessment, adolescents with obesity had higher CDI scores compared to adolescents with overweight and normal BMIs. The implementation of the 1-year lifestyle intervention resulted in a significant reduction in depressive scores in adolescents with obesity and overweight compared to the initial assessment.

### 3.2. Predictors of Depressive and Anxiety Symptomatology

Possible predictors of symptoms of anxiety and depression were investigated through the application of a standard forward multiple stepwise linear regression model (Table S5). Among the anthropometric parameters (weight, height, BMI, WC, HC, WHR, and WHtR), BMI had a predictive function of anxiety scores in the SCARED at the initial (β: 0.154) and annual (β: 0.087) assessment. Among factors related to the metabolic syndrome (glucose, SBP, WC, triglycerides, HDL), WC (β: 0.122), triglycerides (β: 0.135), and SBP (β: 0.105) at the initial assessment were the best positive predictors of depressive scores in the CDI. Notably, a correlation emerged between factors of glucose metabolism and insulin resistance (glucose, insulin, HbA1C, HOMA-IR), with SHBG (β: −0.108) at the initial assessment being negatively associated with depressive symptomatology in the CDI. Among factors assessing pituitary function (TSH, PRL, LH, FSH, ACTH), the concentration of LH (β: 0.123) and prolactin (β: 0.097) at the initial assessment were positive predictors of depression and anxiety scores, respectively. Furthermore, among peripheral hormonal factors (IGF-1, FT4, DHEAS, E2, testosterone, cortisol), testosterone concentration at the first assessment was a negative predictor of depressive and anxiety symptomatology, both at the first assessment and after one year of intervention. In the same grouping, DHEAS concentration (β: 0.143) and IGF-I (β: 0.121) were positively associated with depression scores at the initial assessment, and cortisol concentration (β: 0.101) was positively associated with anxiety symptomatology at the initial assessment.

## 4. Discussion

In the present study, we investigated the effect of a personalized lifestyle intervention on depressive and anxiety symptoms, and their effect and interaction with cardiometabolic parameters in children and adolescents with overweight and obesity before and after the intervention. We demonstrated that following the intervention, both anxiety symptom scores decreased independently of the BMI and depressive symptom scores were improved in the obesity group. During adolescence, the decrease in depressive and anxiety symptomatology proved successful, while in female participants, a reduced response to the intervention was observed. Moreover, cardiometabolic risk factors improved following intervention. Insulin resistance and metabolic syndrome parameters, cortisol, PRL, and LH concentrations were positive predictors for depression and anxiety prior to intervention, while testosterone was a negative predictor before and after the intervention. These findings suggest that the implementation of such an intervention may alleviate not only the cardiometabolic risk but also the depressive and anxiety symptomatology in youth with and without excess adiposity. The improvement in mental health is likely to be mediated by an improvement in energy metabolism with a subsequent improvement in neuroinflammation owing to lifestyle changes.

To begin with, prior to the intervention, no statistically significant difference between anxiety and depression scores and BMI categories was noted, while in subjects with a normal BMI, a decreased—although non-significant—depressive symptom score was found. However, in previous studies, excess adiposity has been associated with increased depressive and anxious moods in the pediatric population [12,45,46]. In a recent meta-analysis that included 22 studies and 143,603 children, the prevalence of depression in children with obesity was 10.4%, with this population having a 1.32-fold higher (95% CI 1.17 to 1.50) likelihood of developing depression compared with children with a normal BMI [47]. It is also worth noting that meta-analysis data suggest a bidirectional association between depression and obesity [46]. The risk difference in depression in early adolescence leading to obesity was 3% higher than for obesity leading to depression. Regarding feelings of anxiety, results from a study of 12,507 children and adolescents showed a higher risk of anxiety and depression in girls and boys with obesity compared with the general population [48]. In Greece, research data show that children with obesity were almost three times more likely to report anxiety and 3.5 times more likely to report depression than children with a normal BMI [49].

Furthermore, a predominant characteristic of excess adiposity in childhood and adolescence is the increased cardiometabolic risk throughout life. Odds of hypertension, hypercholesterolemia, and fasting hyperglycemia progressively double with each increase in the BMI category from normal to overweight to obesity classes I-III [50]. In accordance, participants initially presented central obesity, a distribution of adipose tissue which has been associated with increased morbidity, metabolic imbalance, and significantly higher inflammatory markers, reflecting the chronic low-grade inflammatory process of obesity [51].

Given the emerging problem of childhood obesity and its comorbidities [52], holistic interventions aimed both at physical and mental well-being are of paramount importance. The major finding of the present intervention is the successful improvement in depressive and anxiety symptoms not only in children and adolescents with obesity but in all participants as well. In youth with obesity, research data have already shown the favorable effect of lifestyle interventions in mental health improvement [53]. A meta-analysis by Jebeile H. et al. of 36 interventions showed a reduction in depressive symptomatology, which was maintained for 6–16 months after the initial assessment in 11 studies [53], as well as an amelioration in anxiety symptoms after the interventions (10 studies) and at follow-up (four studies) [53]. Notably, a longer duration of interventions was associated with a greater reduction in anxiety [53]. The accepted minimum duration limit for successful obesity lifestyle interventions in the literature is 6 months [54]. In our study, we applied a long intervention lasting 12 months, which proved to be effective in reducing anxiety. Meanwhile, higher BMI z-scores have been associated with a greater reduction in depression following an intervention [53,55]. In accordance, in the present study, a statistically significant improvement in depressive symptomatology was noted in subjects with a high BMI in the obesity group. Focusing on the frequency of follow-up during an intervention, regular evaluations (weekly or every 15 days) provide the greatest alleviation of depressive moods [53,55]. The present intervention was designed based on this principle, with a structured, systematic (monthly in the obesity group, 2-month intervention in the overweight group, and a 3-month intervention in the normal-BMI group), and individualized follow-up to the needs of each child and his/her family. Overall, a greater reduction in depressive symptoms is positively associated with BMI, while regular follow-up and a long duration of interventions have proven beneficial in combating depression and anxiety, respectively [53]. These findings may raise awareness in healthcare authorities and professionals toward designing effective medical interventions to prevent and manage childhood obesity and its comorbidities.

The present 1-year intervention was also successful in ameliorating anthropometric parameters, cardiovascular risk factors, lipid and glucose metabolism, and non-specific inflammatory markers. Additional studies, which have implemented the presented intervention, have also proven successful in ameliorating dietary habits, clinical and body composition indices of obesity, inflammatory markers, and cardiometabolic risk factors [25,26,27,28,56,57,58], as well as telomere length, an indicator of biological aging and cardiometabolic risk [28]. On the same note, a systematic review of 23 lifestyle interventions, associated BMI and/or BMI z-score reduction with diet and physical activity, the participation of a dietitian/nutritionist and physician in the treatment group, and the longer duration of interventions led to a reduction in cardiovascular risk factors [54]. These principles have been applied in our intervention. Interestingly, a decrease in T3 and FT4 concentrations was also observed following the intervention in participants with obesity. In agreement with previous studies, in euthyroid children with obesity, thyroid hormones are associated with cardiometabolic risk and their changes after interventions are interrelated with changes in cardiometabolic markers, such as glucose and lipid profiles [57,59].

Considering the pathophysiologic mechanisms associating depression, anxiety, and obesity, it is important to note that the overconsumption of fat and sugar, as well as a sedentary lifestyle, lead to the development of excess adiposity, inflammation, and metabolic dysfunction [19]. Excessive visceral fat accumulation leads to immune cell infiltration and cytokine secretion. Therefore, a chronic inflammatory state develops that favors metabolic risk (e.g., insulin and leptin resistance, hypertension), thereby contributing to neurovascular dysfunction (including impaired integrity of the blood–brain barrier), neuroinflammation, and impaired neuroplasticity in mood networks. Interestingly, increased concentrations of inflammation markers, such as CRP, may be used as predictors of depression onset. Moreover, changes in brain composition and lipid (saturated versus unsaturated) and sugar (glucose/fructose) handling, which is associated with obesity-inducing diets, are also implicated in neuroimmunological activation and changes in brain structure and connectivity. Moreover, dysregulation of the hypothalamic–pituitary–adrenal axis is implicated in mood disorders of obesity, with a vicious cycle of cortisol-induced palatable food intake, visceral fat accumulation, and temporary relief with downregulation of the axis taking place. However, the role of cortisol in obesity-associated depression and anxiety is complex and an inter-person variability in glucocorticoid sensitivity may affect those responses [19]. In addition, prolactin, which also acts as a stress hormone, has been associated with multiple psychiatric disorders, such as major depression, paranoid ideation, anxiety, hostility, and somatization. Increased prolactin concentrations lead to a metabolic imbalance with excess adiposity accumulation, lipid and glucose dysregulation, and impairment in dopaminergic pathways [60]. In the present study, the identified predictive role of cardiometabolic risk factors, insulin resistance markers, and cortisol and prolactin concentration in anxiety and depression symptomatology could potentially reflect the above pathophysiologic mechanisms.

Furthermore, in agreement with the literature, the negative predictive role of testosterone concentration in depressive and anxiety symptom development was shown. An elevated depression and anxiety risk upon low testosterone concentrations has been reported in both males and females [61,62,63], with some authors associating it with specific somatic complaints of depression, such as appetite disturbance [64]. Accordingly, two systematic reviews have associated testosterone replacement therapy with the alleviation of depression in males [65,66]. The testosterone antidepressant and anxiolytic benefits in males, females, and rodents are mediated by its organizational and/or activational effects, with the MAPK pathway in the hippocampus exercising a key role [61]. Of particular interest during childhood is the hypogonadal state in males with obesity during all pubertal stages, which is characterized by lower total testosterone concentrations as a result of the insulin resistance-associated reduction in SHBG and the aromatization of testosterone to estradiol in adipose tissue, resulting in low free testosterone concentrations. In human observational studies, aerobic exercise leads to an increase in total testosterone concentrations in early pubertal boys with obesity and enhances anti-oxidation, possibly through the involvement of the maturing hypothalamic–pituitary–testicular (HPT) axis as puberty progresses [67]. It is worth noting that following the 1-year lifestyle intervention, testosterone concentrations significantly increased in children with overweight and obesity. This increase may be interpreted by the pubertal development in participants, the male representation in the sample, or a beneficial role of physical exercise, contributing to depression and anxiety alleviation. However, elucidating such complex interactions is a challenge and demands further research. On the other hand, limited evidence has associated high testosterone concentrations with depression in both sexes, which was not confirmed in our study [64,68,69].

Of particular interest is the lack of an association between the change in depressive symptoms and the improvement in the BMI in lifestyle interventions in the literature [53]. Given that lifestyle interventions address broader aspects of health, it is possible that changes in depression and anxiety are mediated by changes in dietary intake and/or other specific components of the interventions, such as exercise, rather than by weight loss itself [53]. Overall, healthy dietary interventions may lead to improvements in energy and glucose metabolism, a reduction in cardiometabolic risk factors and cortisol concentration, and a subsequent improvement in neuroinflammation [53]. In terms of nutritional parameters, a healthy dietary pattern has been shown to be successful in reducing not only adipose tissue but also inflammation and mental health disorders [70], whereas unhealthy feeding patterns (saturated and trans fats, high-glycemic-index carbohydrates) have been associated with increased levels of inflammation, depression, and anxiety [71]. The contribution of regular physical activity is also important in combating anxiety and depression in the pediatric population [72,73]. Meta-analysis data suggest that interventions which include a structured exercise program show greater reductions in anxiety management than simple physical activity training [53]. Our multidisciplinary lifestyle intervention was associated with improvement in BMI, as well as cardiovascular risk factors and inflammatory markers. These findings suggest that the alleviation of anxiety and depression symptomatology may be attributed to the improvement in energy metabolism by other components of the intervention, except BMI reduction.

Exploring the effect of gender at the initial assessment, boys with obesity showed increased depression and anxiety symptoms compared to their overweight or normal BMI counterparts. Nevertheless, at the annual assessment, male participants showed a significant reduction in depression scores in contrast to female participants, who showed a reduced response to the intervention. Indeed, meta-analysis data suggest a bidirectional association between depression and obesity, which is stronger for adolescent females [46,47]. On that account, research data show that among girls with obesity, the odds of depression were 1.44-fold (95% CI 1.20 to 1.72) higher compared with girls with a normal BMI. The prevalence of depression and anxiety disorders is approximately twice as high for women with obesity compared to men, an association that corresponds to a lifetime prevalence regardless of the BMI [74]. This gender disparity observed throughout life may reflect the differences in the neurobiological processes that characterize anxiety and depression, including fear processing, arousal, social avoidance, and learned helplessness in males and females. The organizational and activational effects of sex hormones contribute to such gender differences, such as the earlier pubertal onset in females or the higher leptin concentrations observed in females with major depression compared to males [61]. In parallel, cultural and behavioral factors are in play. Social discrimination, weight stigma, a poor self-perception of body image, and low self-esteem disproportionally affect females, producing stress and enhancing the vicious circle of obesity, depression, and anxiety [75]. Therefore, the need for earlier and ongoing lifestyle interventions in females is emphasized.

Regarding the effect of puberty, adolescents with obesity in this study showed more severe depressive symptoms compared to their pre-adolescent counterparts. In accordance with the progress of puberty, at the initial assessment, LH concentration emerged as a positive predictor of symptoms of depression and anxiety. This affective disorder was significantly improved through the implemented lifestyle intervention. Increasing research evidence supports a close association of anxiety and depression disorders with adolescence [14], as well as a bidirectional association between depression and obesity during adolescence [76].

The advantages of our study include the large sample of patients and the simultaneous study of multiple clinical, hematologic, biochemical, and endocrinologic parameters. At the same time, the successful implementation of a structured multidisciplinary lifestyle intervention of a long duration and with frequent evaluation offers an effective therapeutic approach to the interrelated disorders of obesity, depression, and anxiety. Regarding the study limitations, no data from clinical psychological interviews of subjects with pathological questionnaire scores are available in order to establish a diagnosis of depression or anxiety disorders. Confounding factors, such as dietary micro- and macro- nutrient intake, physical exercise data, social support, and home environment were not analyzed in detail, while the patients were not followed up with after the end of the study to investigate the persistence over time of the beneficial effects of the intervention. Moreover, a gender-specific analysis regarding testosterone concentrations and its negative predictive role in anxiety and depression symptomatology was not performed. Further multicenter studies are essential to decode the pathophysiological mechanisms, which connect excess adiposity, anxiety and depression alleviation following interventions, the underlying alterations in energy metabolism and neuroinflammatory remission, the effect of gender, and the causal relationship of metabolic and hormonal factors. The use of methods, such as functional MRIs, the measurement of inflammatory markers (interleukins, TNF-α) and micro- and macronutrients in food intake, may provide essential information. Finally, the lack of a non-intervention group limits our understanding of the causal effects of the intervention. Therefore, in future research, a randomized controlled trial with a non-intervention group as a control could further help decode the interrelation of lifestyle interventions with anxiety and depression.

## 5. Conclusions

In conclusion, multidisciplinary personalized lifestyle interventions focusing on a healthy diet, good quality of sleep, and regular exercise are effective in reducing BMI and improving cardiometabolic risk factors, as well as anxiety and depressive symptoms in children and adolescents with overweight and obesity. At the same time, the present study highlights the need for earlier and ongoing interventions in females, where the prevalence of anxiety and depression is increased and there is a reduced response to lifestyle interventions. Insulin resistance parameters associated with the metabolic syndrome, cortisol, PRL, LH, and testosterone concentrations play a predictive role in the development of depression and anxiety. The speculated pathophysiological model is the improvement in energy metabolism and subsequent improvement in neuroinflammation through lifestyle changes. Overall, it is of great importance for healthcare systems to implement early on during childhood personalized multidisciplinary lifestyle interventions to prevent and address pediatric obesity in time and to ensure the long-term benefits of physical and psychosocial well-being in children and adolescents with or without excess adiposity.

## Figures and Tables

**Table 1:** Anthropometric (**A**), hematologic (**B**), biochemical (**C**), cardiometabolic risk factor (**D**), endocrinologic (**E**), and psychometric (**F**) parameters in subjects with obesity (n = 307), with overweight (n = 205), with a normal BMI (n = 99), and all subjects (n = 611) at the initial and annual assessment.

**A. Anthropometric Parameters**	**Initial Assessment**					**Annual Assessment**					***p* Between Timepoints**
	**Obesity**	**Overweight**	**Normal BMI**	**All Groups**	***p* Within Baseline**	**Obesity**	**Overweight**	**Normal BMI**	**All Groups**	***p* Within Follow-Up**	
Age (years)	10.43 ± 0.14	10.41 ± 0.16	10. 24 ± 0.25	10.39 ± 0.10	NS	11. 37 ± 0.16 *	11.39 ± 0.18 *	11.22 ± 0.26 *	11.35 ± 0.11 *	NS	**0.01/0.01/0.01/0.01**
Body weight (kg)	63.81 ± 1.09	50.41 ± 0.87 ^#^	39.87 ± 1.25 ^#+^	55.44 ± 0.75	**0.01**	64.46 ± 1.08 *	52.47 ± 0.96 *^#^	43.38 ± 1.36 *^#+^	57.03 ± 0.76 *	**0.01**	**0.01/0.01/0.01/0.01**
Height (cm)	147.55 ± 0.80	144.84 ± 0.88 ^#^	141.62 ± 1.50 ^#^	145.68 ± 0.56	**0.05**	152.78 ± 0.87 *	150.06 ± 0.94 *^#^	146.99 ± 1.51 *^#^	150.93 ± 0.60 *	**0.05**	**0.01/0.01/0.01/0.01**
BMI (kg/m^2^)	28.59 ± 0.23	23.59 ± 0.15 ^#^	19.37 ± 0.28 ^#+^	25.42 ± 0.19	**0.01**	27.11 ± 0.230 *	22.96 ± 0.19 *^#^	19.66 ± 0.30 ^#+^	24.52 ± 0.19 *	**0.01**	**0.01/0.01**/NS/**0.01**
BMI z-score	2.79 ± 0.06	1.30 ± 0.03 ^#^	0.09 ± 0.08 ^#+^	1.86 ± 0.05	**0.01**	2.11 ± 0.06 *	0.92 ± 0.05 *^#^	0.00 ± 0.08 ^#+^	1.37 ± 0.05 *	**0.01**	**0.01**/**0.01**/NS/**0.01**
SBP (mmHg)	115.15 ± 0.72	108.59 ± 0.85 ^#^	105.93 ± 1.10 ^#+^	111.44 ± 0.52	**0.05**	113.55 ± 0.88	108.16 ± 0.98 *^#^	105.91 ± 1.12 ^#^	110.39 ± 0.60 *	**0.01**	NS/**0.05**/NS/**0.01**
DBP (mmHg)	67.47 ± 0.62	64.90 ± 0.76	63. 48 ± 0.89 ^#+^	65.95 ± 0.43	**0.05**	67.14 ± 0.72	64.84 ± 0.80 ^#^	63.61 ± 0.96 ^#^	65.75 ± 0.48	**0.05**	NS/NS/NS/NS
Waist (cm)	88.72 ± 0.69	77.98 ± 0.57 ^#^	67.57 ± 1.07 ^#+^	81.73 ± 0.54	**0.01**	87.42 ± 0.89 *	78.95 ± 0.76 ^#^	68.82 ± 1.12 ^#+^	81.25 ± 0.66	**0.01**	**0.01**/NS/NS/NS
Hip (cm)	95.32 ± 0.75	85.42 ± 0.68 ^#^	76.52 ± 1.13 ^#+^	88.99 ± 0.56	**0.01**	94.91 ± 0.85	86.73 ± 0.83 ^#^	80.42 ± 1.29 *^#+^	89.57 ± 0.63	**0.01**	NS/NS/**0.01**/NS
Waist-to-hip ratio (WHR)	0.94 ± 0.01	0.92 ± 0.01 ^#^	0.89 ± 0.01 ^#+^	0.92 ± 0.00	**0.01**	0.92 ± 0.01	0.91 ± 0.01	0.86 ± 0.01 ^#+^	0.91 ± 0.00	**0.01**	NS/NS/NS/NS
Waist-to-height ratio (WHtR)	0.60 ± 0.00	0.54 ± 0.00 ^#^	0.48 ± 0.01 ^#+^	0.56 ± 0.00	**0.01**	0.57 ± 0.00 *	0.53 ± 0.00 *^#^	0.47 ± 0.001 ^#+^	0.54 ± 0.00 *	**0.01**	**0.01**/**0.01**/NS/**0.01**
**B. Hematologic Parameters**	**Initial Assessment**					**Annual Assessment**					***p* Between Timepoints**
	**Obesity**	**Overweight**	**Normal BMI**	**All Groups**	***p* Within Baseline**	**Obesity**	**Overweight**	**Normal BMI**	**All Groups**	***p* Within Follow-Up**	
ESR (mm)	20.32 ± 0.77	18.02 ± 0.88 ^#^	12.13 ± 0.83 ^#+^	18.17 ± 0.52	**0.05**	18.02 ± 0.81 *	15.62 ± 1.01	11.41 ± 0.98 ^#+^	16.12 ± 0.56 *	0.01	**0.05**/NS/NS/**0.01**
Ferritin (ng/mL)	60.04 ± 2.05	52.88 ± 3.43	47.97 ± 3.28	55.64 ± 1.64	NS	53.46 ± 2.48 *	50.11 ± 3.73 *	39.42 ± 2.31 *^#^	50.00 ± 1.81 *	0.01	**0.05/0.01/0.05/0.05**
**C. Biochemical Parameters**	**Initial Assessment**					**Annual Assessment**					***p* Between Timepoints**
	**Obesity**	**Overweight**	**Normal BMI**	**All Groups**	***p* Within Baseline**	**Obesity**	**Overweight**	**Normal BMI**	**All Groups**	***p* Within Follow-Up**	
Uric acid (mg/dL)	4.88 ± 0.06	4.46 ± 0.06 ^#^	4.03 ± 0.09 ^#+^	4.60 ± 0.04	**0.01**	4.76 ± 0.08 *	4.48 ± 0.08 ^#^	3.95 ± 0.10 ^#+^	4.53 ± 0.05	**0.01**	**0.01**/NS/NS/NS
AST (U/L)	23.31 ± 0.36	23.21 ± 0.48	23.36 ± 0.48	23.29 ± 0.25	NS	21.99 ± 0.41 *	21.78 ± 0.46 *	22.24 ± 0.65	21.96 ± 0.28 *	NS	**0.01**/**0.01**/NS/**0.01**
ALT (U/L)	22.60 ± 0.75	19.05 ± 0.80 ^#^	15.80 ± 0.46 ^#+^	20.29 ± 0.48	**0.01**	20.61 ± 0.71 *	17.77 ± 0.64 *^#^	16.53 ± 0.60 ^#^	18.99 ± 0.43 *	**0.01**	**0.01**/**0.05**/NS/**0.05**
**D. CVD Risk Factor Parameters**	**Initial Assessment**					**Annual Assessment**					***p* Between Timepoints**
	**Obesity**	**Overweight**	**Normal BMI**	**All Groups**	***p* Within Baseline**	**Obesity**	**Overweight**	**Normal BMI**	**All Groups**	***p* Within Follow-Up**	
Cholesterol (mg/dL)	154.72 ± 1.47	158.22 ± 2.13	154.51 ± 2.40	155.85 ± 1.10	NS	154.35 ± 1.67	156.49 ± 2.28	150.22 ± 2.50	154.41 ± 1.21	NS	NS/NS/NS/NS
Triglycerides (mg/dL)	80.61 ± 2.27	77.03 ±5.05	61.36 ± 3.20 ^#+^	76.22 ± 2.11	**0.05**	81.19 ± 2.89	77.85 ± 3.95	59.74 ± 2.47 ^#+^	76.61 ± 2.04	**0.01**	NS/NS/NS/NS
HDL (mg/dL)	48.93 ± 0.64	54.92 ± 0.96 ^#^	60.04 ± 1.29 ^#+^	52.77 ± 0.53	**0.01**	52.56 ± 0.85 *	57.74 ± 1.10 *^#^	61.19 ± 1.51 *^#^	55.71 ± 0.63 *	**0.01**	**0.01/0.01/0.05/0.01**
LDL (mg/dL)	89. 61 ± 1.32	88.96 ± 1.91	82.64 ± 2.00 ^#^	88.23 ± 0.98	**0.05**	86.30 ± 1.38 *	83.90 ± 2.06 *	77.74 ± 2.17 ^#^	84.11 ± 1.05 *	**0.05**	**0.05**/**0.01**/NS/**0.01**
ApoA1 (mg/dL)	135.50 ± 1.16	142.78 ± 1.42 ^#^	149.20 ± 1.98 ^#^	140.20 ± 0.84	**0.05**	139.10 ± 1.42 *	142.71 ± 1.69	146.28 ± 2.39 ^#^	141.48 ± 1.00	**0.05**	**0.05**/NS/NS/NS
ApoB (mg/dL)	75.49 ± 0.95	73.25 ± 1.28	69.06 ± 1.38 ^#+^	73.68 ± 0.68	**0.05**	73.65 ± 1.03	71.18 ± 1.39	68.61 ± 1.49 ^#^	72.00 ± 0.74	**0.01**	NS/NS/NS/NS
Glucose (mg/dL)	80.53 ± 0.47	79.67 ± 0.53	79.35 ± 0.71	80.05 ± 0.32	NS	82.23 ± 0.47 *	82.44 ± 0.60 *	80.94 ± 0.89 *	82.09 ± 0.34 *	NS	**0.01/0.01/0.05/0.01**
HbA1C (%)	5.25 ± 0.01	5.23 ± 0.02	5.12 ± 0.03 ^#+^	5.22 ± 0.01	**0.01**	5.21 ± 0.02 *	5.21 ±0.02	5.11 ± 0.03 ^#+^	5.19 ± 0.01 *	**0.01**	**0.01**/NS/NS/**0.01**
HOMA-IR	3.61 ± 0.14	2.48 ± 0.11 ^#^	1.87 ± 0.11 ^#+^	2.94 ± 0.09	**0.05**	3.59 ± 0.15	2.80 ± 0.16 *^#^	2.24 ± 0.19 ^#+^	3.10 ± 0.10 *	**0.05**	NS/**0.05**/NS/**0.01**
**E. Endocrinologic Parameters**	**Initial Assessment**					**Annual Assessment**					***p* Between Timepoints**
	**Obesity**	**Overweight**	**Normal BMI**	**All Groups**	***p* Within Baseline**	**Obesity**	**Overweight**	**Normal BMI**	**All Groups**	***p* Within Follow-Up**	
TSH (μIU/mL)	2.98 ± 0.09	2.98 ± 0.11	2.97 ± 0.15	2.98 ± 0.06	NS	2.92 ± 0.09	2.95 ± 0.12	2.87 ± 0.19	2.92 ± 0.07	NS	NS/NS/NS/NS
FT4 (mg/dL)	1.12 ± 0.01	1.13 ± 0.01	1.11 ± 0.01	1.12 ± 0.01	NS	1.09 ± 0.01 *	1.08 ± 0.01 *	1.09 ± 0.02	1.09 ± 0.01 *	NS	**0.05**/**0.01**/NS/**0.01**
T3 (ng/dL)	143.75 ± 1.58	138.45 ± 1.76 ^#^	131.69 ± 2.82 ^#^	139.99 ± 1.10	**0.01**	139.00 ± 1.83 *	132.76 ± 1.88 *^#^	126.91 ± 2.71 ^#^	134.91 ± 1.21 *	**0.01**	**0.05**/**0.05**/NS/**0.01**
IGF-I (ng/mL)	282.27 ± 9.00	289.61 ± 10.86	302.91 ± 19.17	288.10 ± 6.58	NS	363.18 ± 13.40 *	347.13 ± 13.16 *	319.21 ± 19.70	350.58 ± 8.66 *	NS	**0.01**/**0.01**/NS/**0.01**
IGBP3 (ng/mL)	5.08 ± 0.06	4.94 ± 0.07	4.79 ± 0.10 ^#^	4.99 ± 0.04	**0.05**	5.39 ± 0.07 *	5.18 ± 0.08 *^#^	4.89 ± 0.12 ^#^	5.24 ± 0.05 *	**0.05**	**0.01**/**0.01**/NS/**0.01**
Δ4 (ng/mL)	0.98 ± 0.06	1.06 ± 0.08	0.94 ± 0.09	1.00 ± 0.04	NS	1.25 ± 0.07 *	1.28 ± 0.09 *	1.34 ± 0.14 *	1.27 ± 0.05 *	NS	**0.01/0.01/0.01/0.01**
Testosterone (ng/mL)	44.37 ± 4.06	42.66 ± 4.93	47.44 ± 10.21	44.31 ± 3.11	NS	77.93 ± 7.72 *	74.22 ± 9.00 *	44.13 ± 10.20 ^#+^	71.19 ± 5.20 *	**0.05**	**0.01**/**0.01**/NS/**0.01**
DHEAS (μg/dL)	116.99 ± 4.55	107.53 ± 5.91	81.47 ± 6.99 ^#+^	107.91 ± 3.26	**0.01**	137.74 ± 5.82 *	123.82 ± 7.06 *	97.37 ± 9.44 *^#+^	126.48 ± 4.11 *	**0.05**	**0.01/0.01/0.01/0.01**
PRL (ng/mL)	12.16 ± 0.44	11.70 ± 0.45	12.21 ± 0.67	12.01 ± 0.29	NS	12.52 ± 0.45 *	12.23 ± 0.54	13.51 ± 1.05	12.58 ± 0.33 *	NS	**0.05**/NS/NS/**0.01**
LH (mUI/mL)	2.05 ± 0.23	2.70 ± 0.37	1.82 ± 0.31	2.23 ± 0.18	NS	2.98 ± 0.30 *	2.99 ± 0.29	3.33 ±0.80 *	3.04 ± 0.22 *	NS	**0.01**/NS/**0.01**/**0.01**
FSH (mUI/mL)	2.47 ± 0.12	2.71 ± 0.16	2.75 ± 0.20	2.60 ± 0.09	NS	3.13 ± 0.13 *	3.22 ± 0.19 *	3.52 ± 0.26 *	3.23 ± 0.10 *	NS	**0.01/0.01/0.01/0.01**
E2 (pg/mL)	12.29 ± 1.17	20.44 ± 3.42	14.23 ± 1.71	15.33 ± 1.32	NS	17.42 ± 1.96 *	20.24 ± 2.98	28.72 ± 5.68 *^#^	20.21 ± 1.69 *	**0.01**	**0.05**/NS/**0.01**/**0.01**
ACTH (pg/mL)	31.03 ± 1.39	28.99 ± 1.79	26.05 ± 1.89 ^#^	29.52 ± 0.97	**0.05**	31.13 ± 1.80	28.52 ± 1.52	31.64 ± 3.24 *	30.34 ± 1.16	NS	NS/NS/**0.05**/NS
Cortisol (μg/dL)	13.57 ± 0.36	14.13 ± 0.44	13.77 ± 0.56	13.79 ± 0.25	NS	13.17 ± 0.37	12.62 ± 0.45 *	13.21 ± 0.66	12.99 ± 0.26 *	NS	NS/**0.01**/NS/**0.01**
PTH (pg/mL)	35.53 ± 0.73	34.26 ± 0.99	37.94 ± 1.59	35.49 ± 0.56	NS	36.47 ± 0.86	35.90 ± 0.96 *	37.34 ± 1.81	36.41 ± 0.61 *	NS	NS/**0.05**/NS/**0.01**
25OHVitD (ng/mL)	21.84 ± 0.54	25.01 ± 0.71 ^#^	27.37 ± 1.14 ^#^	23.78 ± 0.42	**0.01**	25.41 ± 0.66 *	26.57 ± 0.86 *	27.86 ± 1.24	26.20 ± 0.48 *	NS	**0.01**/**0.01**/NS/**0.01**
Insulin (μIU/mL)	18.20 ± 0.64	12.59 ± 0.51 ^#^	9.54 ± 0.51 ^#+^	14.90 ± 0.40	**0.05**	17.40 ± 0.67	13.68 ± 0.76 ^#^	11.19 ± 0.88 ^#^	15.13 ± 0.46	**0.01**	NS/NS/NS/NS
SHBG (nmol/L)	43.38 ± 1.70	55.52 ± 2.12 ^#^	88.37 ± 4.79 ^#+^	55.38 ± 1.54	**0.05**	45.14 ± 1.85	56.80 ± 2.74 ^#^	86.07 ± 5.58 ^#+^	55.73 ± 1.73 *	**0.01**	NS/NS/NS/**0.05**
**F. Psychometric Questionnaires’ Scores**	**Initial Assessment**					**Annual Assessment**					***p* Between Timepoints**
	**Obesity**	**Overweight**	**Normal BMI**	**All Groups**	***p* Within Baseline**	**Obesity**	**Overweight**	**Normal BMI**	**All Groups**	***p* Within Follow-Up**	
CDI	7.89 ± 0.33	7.49 ± 0.39	6.84 ± 0.56	7.59 ± 0.23	NS	7.08 ± 0.33 *	7.75 ± 0.42	6.74 ± 0.55	7.25 ± 0.24	NS	**0.05**/NS/NS/NS
SCARED parent	1.65 ± 0.09	1.59 ± 0.11	1.49 ± 0.15	1.60 ± 0.06	NS	1.24 ± 0.08 *	1.27 ± 0.11 *	1.07 ± 0.11 *	1.22 ± 0.06 *	NS	**0.01/0.05/0.01/0.01**
SCARED child	2.14 ± 0.10	2.05 ± 0.11	1.97 ± 0.16	2.08 ± 0.07	NS	1.55 ± 0.08 *	1.80 ± 0.10	1.75 ± 0.15	1.66 ± 0.06 *	NS	**0.01**/NS/NS/**0.01**

All results are presented as mean ± SE. Subjects were classified as being obese, overweight, or having a normal BMI according to IOTF criteria at the initial assessment. Tables present comparisons among the three groups at both the initial and annual assessment. All measured variables were compared by employing repeated-measures ANOVA. Significant main effects were revealed by the LSD post hoc test. Statistical significance was set at (*p* < 0.05, rounded to 0.05 in the table), while strong significance (*p* < 0.01, rounded to 0.01 in the table) is also noted and highlighted in bold. NS: nonsignificant (*p* > 0.05) difference. * indicates a significant difference between the initial and annual assessment timepoints, respectively. ^+^ indicates significant difference from the overweight group ^#^ indicates a significant difference from the obese group. *p*-values between two timepoints refer to obesity, overweight, and normal BMI, respectively. CDI: Children’s Depression Inventory, SCARED: Screen for Child Anxiety-Related Disorders.

**Table 2:** Comparison of psychometric scores between initial and annual assessment, with categorization according to the threshold of the questionnaires for detection of pathology in all BMI groups.

BMI Category	Initial Assessment	CDI				SCARED CHILD				SCARED PARENT			
		Annual Assessment		Χ^2^	*p*-Value	Annual Assessment		Χ^2^	*p*-Value	Annual Assessment		Χ^2^	*p*-Value
		Normal Score	Pathological Score			Normal Score	Pathological Score			Normal Score	Pathological Score		
**Obesity**	**Normal Score**	255	13	29.51	**0.01**	174	26	13.00	**0.01**	214	20	35.46	**0.01**
	**Pathological Score**	28	12			75	32			44	28		
**Overweight**	**Normal Score**	168	9	33.68	**0.01**	108	26	7.26	**0.01**	135	20	11.90	**0.01**
	**Pathological Score**	16	11			45	26			32	17		
**Normal BMI**	**Normal Score**	87	5	10.28	**0.01**	50	12	3.74	NS	75	3	18.53	**0.01**
	**Pathological Score**	5	3			24	14			14	8		
**All Subjects**	**Normal Score**	509	28	70.95	**0.01**	332	64	23.84	**0.01**	424	45	61.47	**0.01**
	**Pathological Score**	49	26			144	72			90	53		

BMI: body mass index; CDI: Children´s Depression Inventory; SCARED: Screen for Child Anxiety-Related Disorders. All measured qualitative variables were compared using Pearson’s ×2. Statistical significance was set at *p* < 0.05 (rounded to 0.05 in the table), and a strong significance of *p* < 0.01 (rounded to 0.01 in the table) was also noted; they are both indicated in bold. NS: non-significant (*p* > 0.05) difference.

## Data Availability

The data presented in this study are available upon request from the corresponding author. The data are not publicly available due to privacy restrictions.

## Supplementary Materials

The following supporting information can be downloaded at: https://www.mdpi.com/article/10.3390/nu16213710/s1, Table S1: Supplemental laboratory parameters in subjects who are obese (n = 307), overweight (n = 205), have a normal BMI (n = 99), and all subjects (n = 611) at the initial and annual assessment; Table S2: Change in normal and pathologic psychometric questionnaire scores at initial (A) and annual (Β) assessment in all BMI groups (obesity, overweight, normal BMI); Table S3: Change in psychometric parameters at initial and annual assessment in patients with obesity, overweight, a normal BMI, and all participants, categorized by gender; Table S4: Change in psychometric parameters at initial and annual assessment in patients with obesity, overweight, a normal BMI, and all participants, categorized by pubertal stage; Table S5: Standard forward multiple stepwise linear regression model for the association of depressive and anxiety symptoms with anthropometric, cardiometabolic risk factor, glucose metabolism, and endocrinologic parameters.

## References

[1] WHO Obesity and Overweight 2022. https://www.who.int/news-room/fact-sheets/detail/obesity-and-overweight.

[2] Garrido-Miguel M., Cavero-Redondo I., Álvarez-Bueno C., Rodríguez-Artalejo F., Moreno L.A., Ruiz J.R., Ahrens W., Martínez-Vizcaíno V. (2019). Prevalence and Trends of Overweight and Obesity in European Children From 1999 to 2016: A Systematic Review and Meta-analysis. JAMA Pediatr..

[3] Manios I. Dianeosis. February 2022; pp. 40–61. https://www.dianeosis.org/wp-content/uploads/2022/02/obesity_final11022022.pdf.

[4] van der Klaauw A.A., Farooqi I.S. (2015). The hunger genes: Pathways to obesity. Cell.

[5] Vourdoumpa A., Paltoglou G., Charmandari E. (2023). The Genetic Basis of Childhood Obesity: A Systematic Review. Nutrients.

[6] Styne D.M., Arslanian S.A., Connor E.L., Farooqi I.S., Murad M.H., Silverstein J.H., Yanovski J.A. (2017). Pediatric Obesity-Assessment, Treatment, and Prevention: An Endocrine Society Clinical Practice Guideline. J. Clin. Endocrinol. Metab..

[7] Rankin J., Matthews L., Cobley S., Han A., Sanders R., Wiltshire H.D., Baker J.S. (2016). Psychological consequences of childhood obesity: Psychiatric comorbidity and prevention. Adolesc. Health Med. Ther..

[8] Simmonds M., Llewellyn A., Owen C.G., Woolacott N. (2016). Simple tests for the diagnosis of childhood obesity: A systematic review and meta-analysis. Obes. Rev..

[9] Singh M. (2015). Anthropometric measures during infancy and childhood and the risk of developing cardiovascular disease or diabetes mellitus type 2 in later life: A Systematic Review. ICMR Advanced Centre for Evidence Based Child Health PGIMER.

[10] Zhang T., Zhang H., Li Y., Li S., Fernandez C., Bazzano L., He J., Xue F., Chen W. (2017). Long-term Impact of Temporal Sequence from Childhood Obesity to Hyperinsulinemia on Adult Metabolic Syndrome and Diabetes: The Bogalusa Heart Study. Sci. Rep..

[11] Twig G., Yaniv G., Levine H., Leiba A., Goldberger N., Derazne E., Ben-Ami Shor D., Tzur D., Afek A., Shamiss A. (2016). Body-Mass Index in 2.3 Million Adolescents and Cardiovascular Death in Adulthood. N. Engl. J. Med..

[12] Reilly J.J., Kelly J. (2011). Long-term impact of overweight and obesity in childhood and adolescence on morbidity and premature mortality in adulthood: Systematic review. Int. J. Obes..

[13] Park M.H., Falconer C., Viner R.M., Kinra S. (2012). The impact of childhood obesity on morbidity and mortality in adulthood: A systematic review. Obes. Rev. Off. J. Int. Assoc. Study Obes..

[14] Quek Y.H., Tam W.W.S., Zhang M.W.B., Ho R.C.M. (2017). Exploring the association between childhood and adolescent obesity and depression: A meta-analysis. Obes. Rev. Off. J. Int. Assoc. Study Obes..

[15] Blakemore S.J. (2019). Adolescence and mental health. Lancet.

[16] Poobalan A., Aucott L. (2016). Obesity Among Young Adults in Developing Countries: A Systematic Overview. Curr. Obes. Rep..

[17] Xu C., Miao L., Turner D., DeRubeis R. (2023). Urbanicity and depression: A global meta-analysis. J. Affect. Disord..

[18] SAMHSA, Center for Behavioral Health Statistics and Quality 2023 National Survey on Drug Use and Health. https://www.samhsa.gov/data/report/2023-nsduh-detailed-tables.

[19] Fulton S., Décarie-Spain L., Fioramonti X., Guiard B., Nakajima S. (2022). The menace of obesity to depression and anxiety prevalence. Trends Endocrinol. Metab. TEM.

[20] Tarantino G., Cataldi M., Citro V. (2022). Could Alcohol Abuse and Dependence on Junk Foods Inducing Obesity and/or Illicit Drug Use Represent Danger to Liver in Young People with Altered Psychological/Relational Spheres or Emotional Problems?. Int. J. Mol. Sci..

[21] Prevention-CDC-Centers for Disease Control and Prevention Data and Statistics on Children’s Mental Health. https://www.cdc.gov/children-mental-health/data-research/?CDC_AAref_Val=https://www.cdc.gov/childrensmentalhealth/data.html.

[22] Cole T.J., Bellizzi M.C., Flegal K.M., Dietz W.H. (2000). Establishing a standard definition for child overweight and obesity worldwide: International survey. BMJ (Clin. Res. Ed.).

[23] Cole T.J., Flegal K.M., Nicholls D., Jackson A.A. (2007). Body mass index cut offs to define thinness in children and adolescents: International survey. BMJ (Clin. Res. Ed.).

[24] Cole T.J., Lobstein T. (2012). Extended international (IOTF) body mass index cut-offs for thinness, overweight and obesity. Pediatr. Obes..

[25] Tragomalou A., Moschonis G., Kassari P., Papageorgiou I., Genitsaridi S.M., Karampatsou S., Manios Y., Charmandari E. (2020). A National e-Health Program for the Prevention and Management of Overweight and Obesity in Childhood and Adolescence in Greece. Nutrients.

[26] Genitsaridi S.M., Giannios C., Karampatsou S., Papageorgiou I., Papadopoulos G., Farakla I., Koui E., Georgiou A., Romas S., Terzioglou E. (2020). A Comprehensive Multidisciplinary Management Plan Is Effective in Reducing the Prevalence of Overweight and Obesity in Childhood and Adolescence. Horm. Res. Paediatr..

[27] Karampatsou S.I., Paltoglou G., Genitsaridi S.M., Kassari P., Charmandari E. (2022). The Effect of a Comprehensive Life-Style Intervention Program of Diet and Exercise on Four Bone-Derived Proteins, FGF-23, Osteopontin, NGAL and Sclerostin, in Overweight or Obese Children and Adolescents. Nutrients.

[28] Paltoglou G., Raftopoulou C., Nicolaides N.C., Genitsaridi S.M., Karampatsou S.I., Papadopoulou M., Kassari P., Charmandari E. (2021). A comprehensive, multidisciplinary, personalized, lifestyle intervention program is associated with increased leukocyte telomere length in children and adolescents with overweight and obesity. Nutrients.

[29] Conway J.M., Ingwersen L.A., Moshfegh A.J. (2004). Accuracy of dietary recall using the USDA five-step multiple-pass method in men: An observational validation study. J. Am. Diet. Assoc..

[30] CNPP (2011). U. My Plate Background. https://www.myplate.gov/.

[31] Van Horn L. (2010). Development of the 2010 US Dietary Guidelines Advisory Committee Report: Perspectives from a registered dietitian. J. Am. Diet. Assoc..

[32] Paruthi S., Brooks L.J., D’Ambrosio C., Hall W.A., Kotagal S., Lloyd R.M., Malow B.A., Maski K., Nichols C., Quan S.F. (2016). Consensus Statement of the American Academy of Sleep Medicine on the Recommended Amount of Sleep for Healthy Children: Methodology and Discussion. J. Clin. Sleep. Med. JCSM Off. Publ. Am. Acad. Sleep. Med..

[33] Chiotis D., Krikos X., Tsiftis G., Hatzisymeaon M., Maniati-Christidi M., Dacou-Voutetakis C. (2004). Body mass index and prevalence of obesity in subjects of Hellenic origin aged 0–18 years, living in the Athens area. Ann. Clin. Pediatr. Univ. Atheniensis.

[34] WHO (2008). Waist Circumference and Waist-Hip Ratio: Report of a WHO Expert Consultation.

[35] Stockings E., Degenhardt L., Lee Y.Y., Mihalopoulos C., Liu A., Hobbs M., Patton G. (2015). Symptom screening scales for detecting major depressive disorder in children and adolescents: A systematic review and meta-analysis of reliability, validity and diagnostic utility. J. Affect. Disord..

[36] Sun S., Wang S. (2015). The Children’s Depression Inventory in worldwide child development research: A reliability generalization study. J. Child Fam. Stud..

[37] American Psychiatric Association (2013). Diagnostic and Statistical Manual of Mental Disorders (DSM-5®).

[38] Kovacs M. (1992). Children’s Depression Inventory: Manual/ Multi-Health Systems.

[39] Giannakopoulos G., Kazantzi M., Dimitrakaki C., Tsiantis J., Kolaitis G., Tountas Y. (2009). Screening for children’s depression symptoms in Greece: The use of the Children’s Depression Inventory in a nation-wide school-based sample. Eur. Child Adolesc. Psychiatry.

[40] Hale W.W., Crocetti E., Raaijmakers Q.A., Meeus W.H. (2011). A meta-analysis of the cross-cultural psychometric properties of the Screen for Child Anxiety Related Emotional Disorders (SCARED). J. Child Psychol. Psychiatry.

[41] Birmaher B., Brent D.A., Chiappetta L., Bridge J., Monga S., Baugher M. (1999). Psychometric properties of the Screen for Child Anxiety Related Emotional Disorders (SCARED): A replication study. J. Am. Acad. Child Adolesc. Psychiatry.

[42] Runyon K., Chesnut S.R., Burley H. (2018). Screening for childhood anxiety: A meta-analysis of the screen for child anxiety related emotional disorders. J. Affect. Disord..

[43] Essau C.A., Anastassiou-Hadjicharalambous X., Muñoz L.C. (2013). Psychometric Properties of the Screen for Child Anxiety Related Emotional Disorders (SCARED) in Cypriot Children and Adolescents. Eur. J. Psychol. Assess..

[44] Stavrou S., Nicolaides N.C., Papageorgiou I., Papadopoulou P., Terzioglou E., Chrousos G.P., Darviri C., Charmandari E. (2016). The effectiveness of a stress-management intervention program in the management of overweight and obesity in childhood and adolescence. J. Mol. Biochem..

[45] Rao W.W., Zhang J.W., Zong Q.Q., An F.R., Ungvari G.S., Balbuena L., Yang F.Y., Xiang Y.T. (2019). Prevalence of depressive symptoms in overweight and obese children and adolescents in mainland China: A meta-analysis of comparative studies and epidemiological surveys. J. Affect. Disord..

[46] Mannan M., Mamun A., Doi S., Clavarino A. (2016). Prospective Associations between Depression and Obesity for Adolescent Males and Females- A Systematic Review and Meta-Analysis of Longitudinal Studies. PLoS ONE.

[47] Sutaria S., Devakumar D., Yasuda S.S., Das S., Saxena S. (2019). Is obesity associated with depression in children? Systematic review and meta-analysis. Arch. Dis. Child.

[48] Lindberg L., Hagman E., Danielsson P., Marcus C., Persson M. (2020). Anxiety and depression in children and adolescents with obesity: A nationwide study in Sweden. BMC Med..

[49] Pervanidou P., Bastaki D., Chouliaras G., Papanikolaou K., Laios E., Kanaka-Gantenbein C., Chrousos G.P. (2013). Circadian cortisol profiles, anxiety and depressive symptomatology, and body mass index in a clinical population of obese children. Stress.

[50] Li L., Pérez A., Wu L.T., Ranjit N., Brown H.S., Kelder S.H. (2016). Cardiometabolic Risk Factors among Severely Obese Children and Adolescents in the United States, 1999–2012. Child. Obes..

[51] Liu R., Nikolajczyk B.S. (2019). Tissue Immune Cells Fuel Obesity-Associated Inflammation in Adipose Tissue and Beyond. Front. Immunol..

[52] Sahoo K., Sahoo B., Choudhury A.K., Sofi N.Y., Kumar R., Bhadoria A.S. (2015). Childhood obesity: Causes and consequences. J. Fam. Med. Prim. Care.

[53] Jebeile H., Gow M.L., Baur L.A., Garnett S.P., Paxton S.J., Lister N.B. (2019). Association of Pediatric Obesity Treatment, Including a Dietary Component, with Change in Depression and Anxiety: A Systematic Review and Meta-analysis. JAMA Pediatr..

[54] Bondyra-Wiśniewska B., Myszkowska-Ryciak J., Harton A. (2021). Impact of Lifestyle Intervention Programs for Children and Adolescents with Overweight or Obesity on Body Weight and Selected Cardiometabolic Factors—A Systematic Review. Int. J. Environ. Res. Public Health.

[55] Stice E., Shaw H., Bohon C., Marti C.N., Rohde P. (2009). A meta-analytic review of depression prevention programs for children and adolescents: Factors that predict magnitude of intervention effects. J. Consult. Clin. Psychol..

[56] Karampatsou S.I., Genitsaridi S.M., Michos A., Kourkouni E., Kourlaba G., Kassari P., Manios Y., Charmandari E. (2021). The Effect of a Life-Style Intervention Program of Diet and Exercise on Irisin and FGF-21 Concentrations in Children and Adolescents with Overweight and Obesity. Nutrients.

[57] Ramouzi E., Sveroni K., Manou M., Papagiannopoulos C., Genitsaridi S.M., Tragomalou A., Vourdoumpa A., Koutaki D., Paltoglou G., Kassari P. (2024). The Impact of Thyroid Hormones on Cardiometabolic Risk in Children and Adolescents with Obesity, Overweight and Normal Body Mass Index (BMI): A One-Year Intervention Study. Nutrients.

[58] Ioannou G., Petrou I., Manou M., Tragomalou A., Ramouzi E., Vourdoumpa A., Genitsaridi S.-M., Kyrkili A., Diou C., Papadopoulou M. (2024). Dietary and Physical Activity Habits of Children and Adolescents before and after the Implementation of a Personalized, Intervention Program for the Management of Obesity. Nutrients.

[59] Reinehr T., Andler W. (2002). Thyroid hormones before and after weight loss in obesity. Arch. Dis. Child..

[60] Pirchio R., Graziadio C., Colao A., Pivonello R., Auriemma R.S. (2022). Metabolic effects of prolactin. Front. Endocrinol..

[61] McHenry J., Carrier N., Hull E., Kabbaj M. (2014). Sex differences in anxiety and depression: Role of testosterone. Front. Neuroendocr..

[62] McIntyre R.S., Mancini D., Eisfeld B.S., Soczynska J.K., Grupp L., Konarski J.Z., Kennedy S.H. (2006). Calculated bioavailable testosterone levels and depression in middle-aged men. Psychoneuroendocrinology.

[63] Giltay E.J., Enter D., Zitman F.G., Penninx B.W., van Pelt J., Spinhoven P., Roelofs K. (2012). Salivary testosterone: Associations with depression, anxiety disorders, and antidepressant use in a large cohort study. J. Psychosom. Res..

[64] Määttänen I., Gluschkoff K., Komulainen K., Airaksinen J., Savelieva K., García-Velázquez R., Jokela M. (2021). Testosterone and specific symptoms of depression: Evidence from NHANES 2011–2016. Compr. Psychoneuroendocrinol..

[65] Walther A., Breidenstein J., Miller R. (2019). Association of Testosterone Treatment with Alleviation of Depressive Symptoms in Men: A Systematic Review and Meta-analysis. JAMA Psychiatry.

[66] Zarrouf F.A., Artz S., Griffith J., Sirbu C., Kommor M. (2009). Testosterone and depression: Systematic review and meta-analysis. J. Psychiatr. Pr..

[67] Paltoglou G., Avloniti A., Chatzinikolaou A., Stefanaki C., Papagianni M., Papassotiriou I., Fatouros I.G., Chrousos G.P., Kanaka-Gantenbein C., Mastorakos G. (2019). In early pubertal boys, testosterone and LH are associated with improved anti-oxidation during an aerobic exercise bout. Endocrine.

[68] Booth A., Johnson D.R., Granger D.A. (1999). Testosterone and men’s depression: The role of social behavior. J. Health Soc. Behav..

[69] Weber B., Lewicka S., Deuschle M., Colla M., Heuser I. (2000). Testosterone, androstenedione and dihydrotestosterone concentrations are elevated in female patients with major depression. Psychoneuroendocrinology.

[70] Khalid S., Reynolds S.A., Williams C.M. (2017). Is there an association between diet and depression in children and adolescents? A systematic review. Br. J. Nutr..

[71] Oddy W.H., Allen K.L., Trapp G.S., Ambrosini G.L., Black L.J., Huang R.C., Rzehak P., Runions K.C., Pan F., Beilin L.J. (2018). Dietary patterns, body mass index and inflammation: Pathways to depression and mental health problems in adolescents. Brain Behav. Immun..

[72] Hu S., Li X., Yang L. (2023). Effects of physical activity in child and adolescent depression and anxiety: Role of inflammatory cytokines and stress-related peptide hormones. Front. Neurosci..

[73] Korczak D.J., Madigan S., Colasanto M. (2017). Children’s Physical Activity and Depression: A Meta-analysis. Pediatrics.

[74] Zhao G., Ford E.S., Dhingra S., Li C., Strine T.W., Mokdad A.H. (2009). Depression and anxiety among US adults: Associations with body mass index. Int. J. Obes..

[75] Tronieri J.S., Wurst C.M., Pearl R.L., Allison K.C. (2017). Sex Differences in Obesity and Mental Health. Curr. Psychiatry Rep..

[76] Mühlig Y., Antel J., Föcker M., Hebebrand J. (2016). Are bidirectional associations of obesity and depression already apparent in childhood and adolescence as based on high-quality studies? A systematic review. Obes. Rev..

