# Supplementary material for: Improvement in Symptoms of Depression and Anxiety and Cardiometabolic Risk Factors in Children and Adolescents with Overweight and Obesity Following the Implementation of a Multidisciplinary Personalized Lifestyle Intervention Program
Source: Nutrients. 2024 Oct 30;16(21):3710. doi: 10.3390/nu16213710 (PMC11547602; doi:10.3390/nu16213710)
Supplement: Supplementary file 1 [file nutrients-16-03710-s001.zip › nutrients-3252722-supplementary.pdf]

Supplemental Table S1. Supplemental laboratory parameters in subjects with obesity (n=307), overweight (n=205), normal BMI (n=99) and all subjects (n=611) at initial and annual assessment.

	**Initial Assessment**					**Annual Assessment**					
**Laboratory Parameters**	**Obesity**	**Overweight**	**Normal BMI**	**All Groups**	**P within baseline**	**Obesity**	**Overweight**	**Normal BMI**	**All Groups**	**P within follow-up**	**P between timepoints**
**WBC × 103/μL**	7.46 ± 0.08	7.23 ± 0.14	7.01 ± 0.23	7.31 ± 0.08	NS	7.37 ± 0.12	6.74 ± 0.14*#	6.56 ± 0.16*#	7.02 ± 0.08*	0.01	NS/**0.01/0.01/0.01**
RBC x 1003/μL	5.01 ± 0.03	4.93 ± 0.03	4.98 ± 0.05	4.98 ± 0.01	NS	5.02 ± 0.03	4.96 ± 0.04	5.03 ± 0.05	5.00 ± 0.02	NS	NS
Hb (g/dL)	12.87 ± 0.06	12.78 ± 0.06	12.94 ± 0.10	12.85 ± 0.04	NS	12.98 ± 0.06*	12.89 ± 0.08	13.03 ± 0.11	12.96 ± 0.05*	NS	**0.01**/NS/NS/**0.01**
Hct (%)	40.18 ± 0.16	39.93 ± 0.18	40.45 ± 0.28	40.14 ± 0.1140	NS	40.60 ± 0.18*	40.23 ± 0.24	40.52 ± 0.30	40.46 ± 0.13*	NS	**0.05**/NS/NS/**0.01**
PLT x 103/μL	297.61 ± 3.78	294.28 ± 4.37	285.97 ± 6.37	294.57 ± 2.6120	NS	287.15 ± 4.25*	282.99 ± 4.66*	279.29 ± 7.12*	284.45 ± 2.88*	NS	**0.05/0.01/0.01/0.01**
Folic Acid (ng/mL)	11.15 ± 0.42	10.36 ± 0.33	10.85 ± 0.48	10. 84 ± 0.25	NS	8.60 ± 0.31*	8.59 ± 0.35*	8.97 ± 0.54*	8.66 ± 0.21*	NS	**0.01/0.01/0.01/0.01**
Urea (mg/dL)	28.39 ± 0.37	28.11 ± 0.44	28.86 ± 0.67	28.38 ± 0.26	NS	28.79 ± 0.45	27.63 ± 0.45	27.69 ± 0.73	28.22 ± 0.30	NS	NS/NS/NS/NS
Creatinine (mg/dL)	0.51 ± 0.01	0.51 ± 0.01	0.49 ± 0.01	0.51 ± 0.00	NS	0.54 ± 0.01*	0.54 ± 0.01*	0.53 ± 0.01*	0.54 ± 0.01*	NS	**0.01/0.01/0.01/0.01**
K (mmol/L)	4.39 ± 0.02	4.39 ± 0.02	4.33 ± 0.03#+	4.38 ± 0.01	**0.05**	4.43 ± 0.02	4.40 ± 0.03	4.38 ± 0.03	4.41 ± 0.01	NS	NS/NS/NS/NS
Na (mmol/L)	140. 32 ± 0.09	140.28 ± 0.12	140.66 ± 0.17	140.36 ± 0.06	NS	140.43 ± 0.10	140.15 ± 0.12	140.49 ± 0.18	140.35 ± 0.07	NS	NS/NS/NS/NS
Cl (mmol/L)	102.35 ± 0.23	102.01 ± 0.27	102.65 ± 0.35	102.27 ± 0.16	NS	101.18 ± 0.22*	101.90 ± 0.30	101.24 ± 0.50	101.40 ± 0.17*	NS	**0.01**/NS/NS/**0.05**
γ-GT (U/L)	14.93 ± 0.33	12.86 ± 0.33#	10.95 ± 0.29#+	13.58 ± 0.21	0.01	13.71 ± 0.34*	13.21 ± 0.73	10.50 ± 0.37#+	13.02 ± 0.31*	0.01	0.05/NS/NS/0.05
Albumin (g/dL)	4.62 ± 0.01	4.61 ± 0.02	4.62 ± 0.02	4.62 ± 0.01	NS	4.60 ± 0.01	4.60 ± 0.02	4.62 ± 0.02	4.60 ± 0.01	NS	NS/NS/NS/NS

ALP (U/L)	235.67 ± 4.33	225.42 ± 5.44#	243.75 ± 7.62	233.60 ± 3.11	0.05	224.77 ± 5.26*	220.30 ± 6.46	228.65 ± 9.49	223.88 ± 3.74*	NS	0.01/NS/NS/0.01
PO4 (mmol/L)	4.63 ± 0.03	4.68 ± 0.04	4.71 ± 0.05	4.66 ± 0.02	NS	4.67 ± 0.04	4.67 ± 0.04	4.59 ± 0.07*	4.66 ± 0.03	NS	NS/NS/0.05/NS
Ca (mmol/L)	9.86 ± 0.02	9.83 ± 0.02	9.81 ± 0.03	9.84 ± 0.01	NS	9.75 ± 0.02*	9.73 ± 0.03*	9.71 ± 0.04*	9.74 ± 0.02*	NS	0.01/0.01/0.05/0.01
Lp(a) (mg/dL)	17.63 ± 1.68	14.48 ± 1.41	19.88 ± 2.99	16.96 ± 1.09	NS	16.92 ± 1.73	15.12 ± 1.82	17.59 ± 3.13	16.41 ± 1.17	NS	NS/NS/NS/NS
Anti-TG (IU/mL)	28.80 ± 6.64	27.32 ± 3.18	27.71 ± 4.22#+	28.12 ± 3.54	**0.05**	22.66 ± 1.83	24.15 ± 2.40	32.00 ± 6.24#	24.68 ± 1.59	**0.01**	NS/NS/NS/NS
Anti-TPO (IU/mL)	21.04 ± 4.68	32.20 ± 8.62	30.12 ± 12.47	26.28 ± 4.24	NS	15.31 ± 3.06	24.02 ± 6.03	41.20 ± 19.33#	22.48 ± 4.06	**0.05**	NS/NS/NS/NS

All results are presented as mean ± SE. Subjects were classified as having obesity, overweight, or normal BMI according to IOTF criteria at initial assessment. Tables present comparisons among the three groups at both initial and annual assessment. All measured variables were compared by employing repeated-measures ANOVA. Significant main effects were revealed by the LSD posthoc test. Statistical significance was set at ($p < 0.05$, rounded to 0.05 in Table), while strong significance ($p < 0.01$, rounded to 0.01 in Table) is also noted. NS: nonsignificant ($p > 0.05$) difference. *: Indicates significant difference between initial and annual assessment, timepoints respectively. +: Indicates significant difference from Overweight group #: Indicates significant difference from Obese group. p-values between two timepoints refer to obese, overweight, and normal BMI respectively.

Supplemental Table S2. Change in Normal and Pathologic Psychometric Questionnaire Scores at Initial (A) and Annual (B) assessment in all BMI groups (Obesity, Overweight, Normal BMI).

Evaluation	BMI Category	C.D.I				S.C.A.R.E.D CHILD				S.C.A.R.E.D PARENT			
		Normal Scores	Pathological Scores	X^2	P-value	Normal Scores	Pathological Scores	X^2	P-value	Normal Scores	Pathological Scores	X^2	P-value
**A. Initial Assessment**	**Obesity**	267 (87%)	40 (13%)	1.2	NS	200 (65.1%)	107 (34.9%)	0.4	NS	235 (76.5%)	72 (23.5%)	0.1	NS
	**Overweight**	178 (86.8%)	27 (13.2%)			134 (65.9%)	71 (34.1%)			156 (76.1%)	49 (23.9%)		
	**Normal BMI**	90 (90.9%)	9 (9.1%)			61 (60.6%)	38 (39.4%)			77 (77.8%)	22 (22.2%)		

**B. Annual Assessment**	**Obesity**	282 (91.9%)	25 (8.1%)	0.7	NS	249 (81.1%)	58 (18.9%)	4	NS	257 (83.7%)	50 (16.3%)	2.4	NS
	**Overweight**	184 (89.8%)	21 (10.2%)			153 (74.6%)	52 (25.4%)			168 (82%)	37 (18%)		
	**Normal BMI**	91 (91.9%)	8 (8.1%)			73 (73.7%)	26 (26.3%)			88 (88.9%)	11 (11.1%)		

BMI: Body Mass Index, CDI: Child´s Depression Inventory, SCARED: Screen for Child Anxiety Related Disorders. All measured qualitative variables were compared using Pearson's x2. Statistical significance was set at $p < 0.05$ (rounded to 0.05 in the Table), and a strong significance of $p < 0.01$ (rounded to 0.01 in the Table) was also noted. NS: non-significant ($p > 0.05$) difference.

Supplemental Table S3. Change of psychometric parameters at initial and annual assessment in patients with obesity, overweight, normal BMI and all participants, categorized by gender.

	**Initial Assessment**									**Annual Assessment**									**P between timepoints**	
	**MALE**				**FEMALE**					**MALE**				**FEMALE**						
**Psychometric Questionnaires**	**Obesity**	**Overweight**	**Normal BMI**	**P within baseline**	**Obese**	**Overweight**	**Normal BMI**	**P within baseline**	**P between Genders**	**Obesity**	**Overweight**	**Normal BMI**	**P within follow-up**	**Obese**	**Overweight**	**Normal BMI**	**P within follow-up**	**P between Genders**	**MALE**	**FEMALE**
**C.D.I**	8.01 ± 0.41	7.28 ± 0.54	5.29 ± 0.65#	**0.05**	7.74 ± 0.54	7.67 ± 0.57	7.54 ± 0.75	NS	NS	6.82 ± 0.41*	7.51 ± 0.48	6.55 ± 0.79	NS	7.42 ±0.54	7.96 ±0.67	6.83 ± 0.71	NS	NS	**0.01**/NS/NS	NS
**S.C.A.R.E.D Parent**	1.56 ± 0.11	1.47 ± 0.16	0.90 ± 0.21#	**0.05**	1.76 ± 0.15	1.68 ± 0.16	1.76 ± 0.19	NS	NS/NS/**0.01**	1.09 ± 0.09*	1.15 ± 0.16*	0.65 ± 0.15	NS	1.43 ± 0.13*	1.37 ± 0.16*	1.26 ± 0.14*	NS	**0.05**/NS/NS	**0.01/0.05/**NS	**0.01/0.05/0.01**
**S.C.A.R.E.D Child**	2.07 ± 0.13	1.66 ± 0.15#	1.26 ± 0.23#	**0.01**	2.22 ±0.16	2.38 ± 0.16	2.29 ± 0.20	NS	NS/**0.01/0.05**	1.38 ±0.10*	1.37 ± 0.13	1.00 ± 0.21	NS	1.75 ± 0.13*	2.17 ±0.15#	2.09 ±0.19	0.05	**0.05/0.01/0.01**	**0.01**/NS/NS	**0.01**/NS/NS

NS: nonsignificant (p > 0.05) difference *: Indicates significant difference between initial and annual assessment, timepoints respectively. +: Indicates significant difference from Overweight group #: Indicates significant difference from Obese group. p-values between two timepoints refer to obese, overweight, and normal BMI respectively.

Supplemental Table S4. Change of psychometric parameters at initial and annual assessment in patients with obesity, overweight, normal BMI and all participants, categorized by pubertal stage.

Psychometric Questionnaires	Initial Assessment									Annual Assessment									P between timepoints	
	PREPUBERTAL				PUBERTAL				P between Pubertal stages	PREPUBERTAL				PUBERTAL				P between Pubertal stages	PREPUBERTAL	PUBERTAL
	Obesity	Overweight	Normal BMI	P within baseline	Obese	Overweight	Normal BMI	P within baseline		Obesity	Overweight	Normal BMI	P within follow-up	Obese	Overweight	Normal BMI	P within follow-up			
CDI	7.09 ± 0.41	6.92 ± 0.51	6.12 ± 0.62	NS	8.88 ± 0.53	8.08 ± 0.60	7.54 ± 0.95	NS	**0.01**/NS/NS	6.70 ± 0.41	6.49 ± 0.47	6.09 ± 0.72	NS	7.61 ± 0.54	9.51 ± 0.72 #	7.49 ± 0.84+	**0.05**	NS/**0.01**/NS	NS	**0.05/0.01**/NS
SCARED Parent	1.76 ± 0.11	1.62 ± 0.16	1.57 ± 0.22	NS	1.53 ± 0.15	1.57 ± 0.16	1.42 ± 0.21	NS	NS	1.26 ± 0.11*	1.56 ± 0.17	1.02 ± 0.17*+	**0.05**	1.22 ± 0.12*	0.91 ± 0.14*	1.15 ± 0.16	NS	NS/**0.01**/NS	**0.01**/ NS/**0.01**	**0.01/0.01/** NS
SCARED Child	2.13 ± 0.14	1.96 ± 0.16	2.08 ± 0.26	NS	2.17 ± 0.15	2.13 ± 0.17	1.85 ± 0.21	NS	NS	1.60 ± 0.11	1.77 ± 0.14	1.78 ± 0.23	NS	1.49 ± 0.12*	1.84 ± 0.16*#	1.75 ± 0.22+	**0.05**	NS/**0.01**/NS	**0.01**/NS/NS	**0.05**/NS/NS

NS: nonsignificant (p>0.05) difference *: Indicates significant difference between initial and annual assessment, timepoints respectively. +: Indicates significant difference from Overweight group #: Indicates significant difference from Obese group. p-values between two timepoints refer to obese, overweight, and normal BMI respectively.

Supplemental Table S5. Standard Forward, Multiple Stepwise Linear Regression Model for the association of depressive and anxiety symptoms with anthropometric, cardiometabolic risk factors, glucose metabolism and endocrinologic parameters.

	CDI Initial Assessment	CDI Annual Assessment	SCARED Parent Initial Assessment	SCARED Parent Annual Assessment	SCARED Child Initial Assessment	SCARED Child Annual Assessment	P-Value
**Anthropometric Parameters (Weight, Height, BMI, WC, HC, WHR and WHtR)**							
Weight	β: 0.187	-	-	-	-	-	
Height	-	β: 0.173	β: -0.115	β: -0.179	β: -0.100	β: -0.187	
WHR	-	-	-	-	-	β: -0.087	
BMI	-	-	-	β: 0.087	β: 0.154	-	
**Metabolic Syndrome Parameters (Glucose, SBP, WC, Triglycerides, HDL)**							
WC	β: 0.122	**-**	**-**	**-**	**-**	**-**	
Triglycerides	β: 0.135	**-**	**-**	**-**	**-**	**-**	
SBP	β: 0.105	**-**	**-**	**-**	**-**	**-**	
**Glucose Metabolism and Insulin Sensitivity Parameters (Glucose, Insulin, HbA1C, HOMA-IR)**							**<0.05**
SHBG	β: −0.108	-	-	-	-	-	
**Pituitary Function Parameters (TSH, PRL, LH, FSH, ACTH)**							
LH	β: 0.123	-	-	-	-	-	
PRL	-	-	β: 0.097	-	-	-	
**Peripheral Hormones (IGF-1, fT4, DHEAS, E2, Testosterone, Cortisol)**							
Testosterone	β:-108	β: -105	β:-118	β: -0.112	β: -0.152	β: -0.175	
DHEAS	β: 0.143	-	-	-	-	-	
IGF-I	β: 0.121	-	-	-	--	-	
Cortisol	-	-	β: 0.101	-	-	-	

Abbreviations: ACTH: Adrenocorticotropic hormone, BMI: Body Mass Index, DHEAS: Dehydroepiandrosterone, E2: Estradiol, FSH: Follicle-stimulating hormone, HBA1C: hemoglobin A1c, HC: Hip Circumference, HDL: High-density lipoprotein, HOMA-IR: Homeostasis Model Assessment – Insulin Resistance, IGF-I: Insulin-like growth factor I, LH: Luteinizing Hormone, NS: Non-significant difference, PRL: Prolactin, SHBG: sex hormone binding globulin, SPB: Systolic Blood Pressure, WC: Waist Circumference, WHR: Waist to Hip Ratio, WHtR: Waist to Height R
